# Optimal navigation of a smart active particle: directional and distance sensing

**DOI:** 10.1140/epje/s10189-023-00309-3

**Published:** 2023-06-19

**Authors:** Mischa Putzke, Holger Stark

**Affiliations:** Institut für Theoretische Physik, Technische Universität Berlin, Hardenbergstr. 36, 10623 Berlin, Germany

## Abstract

**Abstract:**

We employ *Q* learning, a variant of reinforcement learning, so that an active particle learns by itself to navigate on the fastest path toward a target while experiencing external forces and flow fields. As state variables, we use the distance and direction toward the target, and as action variables the active particle can choose a new orientation along which it moves with constant velocity. We explicitly investigate optimal navigation in a potential barrier/well and a uniform/ Poiseuille/swirling flow field. We show that *Q* learning is able to identify the fastest path and discuss the results. We also demonstrate that *Q* learning and applying the learned policy works when the particle orientation experiences thermal noise. However, the successful outcome strongly depends on the specific problem and the strength of noise.

**Graphical abstract:**

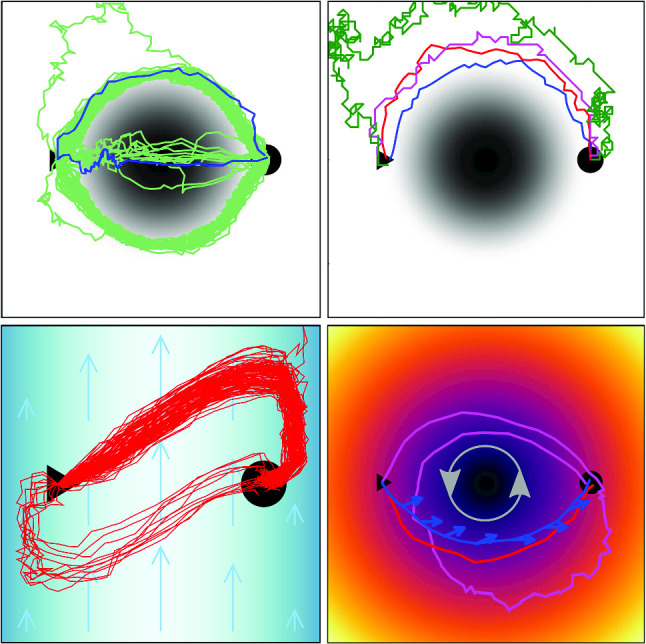

## Introduction

Active matter refers to materials that consist of self-propelled entities such as active particles, artificial microswimmers, and microorganisms, which exhibit versatile collective behavior and dynamic patterns [1–3]. These systems are characterized by their ability to use an internal energy depot or energy from the environment to generate active motion, for example, by deformations [1, 2, 4–8]. Examples of active matter are biological systems including bacteria [9], schools of fish and swarms of birds [10, 11] as well as artificial systems such as suspensions of self-propelled colloids or other synthetic microswimmers [12–14]. Over the years, interest in the control of active motion has increased using external fields [15], in particular, gravitational [16–22], magnetic [23, 24], and flow fields [25–29].

With the ability to specifically manipulate active motion, optimizing the traveled path in a complex environment, for example, by finding the fastest trajectory has come into focus. While optimal search strategies depend on the environment [30], Ref. [31] suggests minimal navigation strategies and in Refs. [32, 33] optimal navigation is achieved by minimizing travel time. This can be done even on curved manifolds such as a sphere [34] and using optimal control theory [35, 36]. Furthermore, the noisy pursuit of a self-steering active particle has been studied [37].

For living organisms, there are many examples where optimal navigation is crucial, such as finding food sources [38, 39] or escaping from predators [40]. While organisms have learned their optimal navigation strategy through evolution, reinforcement learning [41] offers a promising method of training artificial microswimmers to steer optimally toward a target. Applications range from robotics [42, 43] to biology and active matter[14, 33, 44–47]. Reinforcement learning is a type of machine learning where an agent learns a specific task by taking actions in an environment and receiving feedback in the form of rewards. Now, active particles can use this algorithm to learn how to navigate optimally based on their sensory inputs and reward signals. An example are microswimmers that move toward a target by adjusting their orientations along which self-propulsion occurs. In the last years, it has been demonstrated that reinforcement learning is a well suited method for finding optimal navigation solutions in, for example, complex potentials [33, 48, 49], turbulent flows [50–53], as well as chaotic flows [54].

In this article, we employ *Q* learning, a variant of reinforcement learning, so that the agent or microswimmer learns by itself to move on the fastest path from the starting point to the target under the action of forces and flow fields. This is the traditional Zermelo navigation problem [55]. In contrast to our previous work [33], the microswimmer can sense the direction and distance to a target, which we find potentially easier to realize than monitoring the position. The smart active particle first moves deterministically and can control its orientation. We show that the microswimmer with the new state variables is able to identify and navigate on the fastest path in different complex landscapes such as potential barriers and wells as well as uniform, Poiseuille, and swirling flow fields. In addition, we show that learning optimal navigation and applying the learned policy also works under thermal noise, which the particle orientation experiences during training. However, the outcome strongly depends on the specific problem and the strength of noise.

The article is structured as follows. In Sect. 2, we introduce our model, the method of *Q* learning, the choice of state and action variables, and the system parameters. In Sect. 3, we present our results of the potential barrier/well and the uniform/Poiseuille/swirling flow fields. We close with conclusions.

**Fig. 1:**
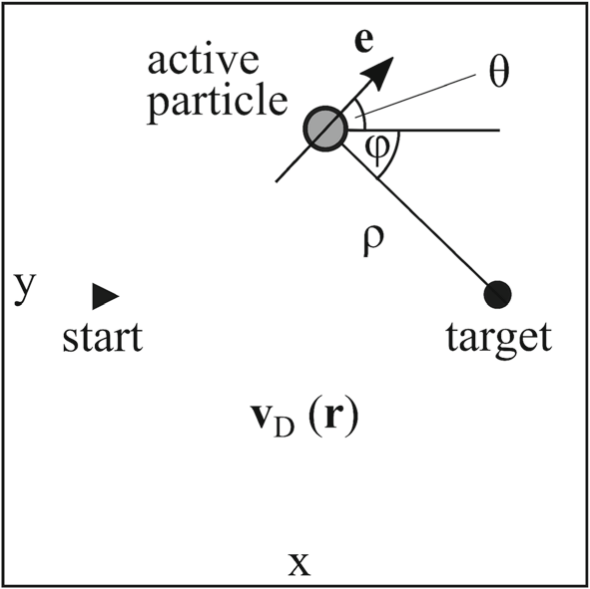
The active particle with orientation unit vector $\textbf{e}$ is trained to move from the starting to target position on the fastest path crossing a region with a prescribed drift-velocity field $\textbf{v}_D(\textbf{r})$. To locate its position, the particle can sense the direction angle $\varphi$ and distance $\rho$ to the target

## Model

### Equations of motion

Our goal is to train the microswimmer such that it optimizes its travel time moving in two dimensions from a starting to a target position. Along its path, it experiences an additional drift-velocity field, which represents some complex environment (see Fig. 1). We simply model the microswimmer as an active particle with negligible inertia that moves with speed $v_0$ along the direction $\textbf{e}=(\cos \theta ,\sin \theta )$, where $\theta$ is the angle with respect to the *x* axis (see Fig. 1). Rescaling all velocities by $v_0$, the space-dependent total velocity of the active particle is

1 $$\begin{aligned} \textbf{v} = \textbf{e} + \textbf{v}_D \, . \end{aligned}$$

We assume that the active particle can control its orientation $\textbf{e}$ in order to find the optimal travel time. For this, every time $\Delta t$ it senses its state and uses *Q* learning to set its new orientation $\textbf{e}$ as we explain in Sects. 2.2 and 2.3. Furthermore, we do not include any external torque. However, for the drift-velocity field $\textbf{v}_D(\textbf{r})$ we will consider one type of flow field (Poiseuille flow) with nonzero vorticity, which rotates the particle orientation with angular velocity $\omega _D= | \text {curl} \textbf{v}_D | /2$. Thus, during time step $\Delta t$, the particle’s orientation evolves according to $\dot{\theta } = \omega _D$.

The optimization of the travel time can also be formulated as a typical variational principle. The time to travel from the starting position at $\textbf{r}_i$ to the target position at $\textbf{r}_f$ is

2 $$\begin{aligned} T = \int _{\textbf{r}_i}^{\textbf{r}_f} \textrm{d}t = \int _{\textbf{r}_i}^{\textbf{r}_f} \frac{\textrm{d}s}{v} \, , \end{aligned}$$

where $v= \vert \textbf{v} \vert$. We parametrize the particle path $\textbf{r}(s)$ with the arclength *s*, introduce the unit tangent along the path, $\textbf{t}= \textrm{d} \textbf{r} / \textrm{d}s$, and write the total velocity as $\textbf{v} = v \textbf{t}$. Taking the square of Eq. (1) and solving for *v*, we obtain

3 $$\begin{aligned} v = \textbf{t} \cdot \textbf{v}_D + \sqrt{1 - [\textbf{v}_D^2 - (\textbf{t} \cdot \textbf{v}_D)^2]} \, . \end{aligned}$$

With this the variation of the travel time, $\delta T = 0$, in order to find a minimum can be formulated. Typically, the minimum has to be calculated numerically and only in special cases an analytic solution is possible.

Besides treating the active particle fully deterministically, we will also explore how thermal fluctuations influence the learning of the optimal path and when applying the learned optimal policy. Since we will work at large Péclet numbers, where translational noise can be discarded, we concentrate on thermal noise in the orientation. Thus, we use the model of an active Brownian particle and write down an overdamped Langevin equation for the time derivative of the orientation angle $\theta$. Including the nonzero vorticity for one type of flow field, the Langevin equation becomes

4 $$\begin{aligned} \dot{\theta } = \omega _D+ \sqrt{D_R L/v_0}\ \eta \, . \end{aligned}$$

Here, $\eta$ represents standard Gaussian white noise with zero mean $\langle \eta (t) \rangle = 0$ and unit variance $\langle \eta (t) \eta (t') \rangle =\delta (t-t')$. The thermal rotational diffusion coefficient $D_R$ ensures the validity of the fluctuation-dissipation theorem. Time is rescaled by the characteristic time scale $L/v_0$ and *L* is a typical length, which we choose as the distance between starting and target position.

Now, to include rotational noise in the training process and when applying the learned policy, we need to numerically integrate Eq. (4). At each step of *Q* learning, the orientation angle is set to a value $\theta$ as explained in Sect. 2.3. Starting from this value, the new orientation angle $\theta ^{\textrm{new}}$ after time step $\Delta t$ becomes

5 $$\begin{aligned} \theta ^{\textrm{new}} = \theta + \omega _D \Delta t + \sqrt{(D_R L/v_0) \Delta t}\ W_{\theta } \, , \end{aligned}$$

where $\sqrt{\Delta t} W_\theta$ represents the increment of a Wiener process and $W_\theta$ is a random number with zero mean and unit variance $\langle W_\theta ^2 \rangle$. The new angle $\theta ^{\textrm{new}}$ is then used to perform a step of the active particle, which is further processed as explained in Sect. 2.3.

With the formulated model, we fully describe the dynamics of our microswimmer. Now, we can start using *Q* learning to train the swimmer such that it finds the optimal path in various drift-velocity fields.

### *Q* learning

In order for an active particle to learn the fastest path from an initial position to an assigned target, while moving in an external drift-velocity field, the method of tabular *Q* learning can be applied [41] Here, one first creates a *Q* table that stores a *Q* value for each pair of state (e.g., position of the particle) and possible action variables (e.g., movement in a certain direction). The *Q* value represents the expected reward accumulated during a sequence of steps when starting from a state-action pair. Thus, it not only considers the immediate reward but future rewards as well.

To start the *Q* learning algorithm, the active particle is placed at the initial position and moves under the influence of the drift-velocity field in a series of steps. Before it performs an action, the *Q* values of the possible actions in the current state are checked and the action with the highest *Q* value is selected. It represents the highest expected accumulated reward. After each action, the corresponding *Q* value for the state-action pair is adjusted as reported below. When the active particle reaches the target, the first training episode is completed. A new episode begins with the same initial state, and the process repeats until the *Q* matrix of the agent converges to a stable solution. This means that the agent has learned the optimal policy, which for each of the states gives the optimal action such that the total reward accumulated by the agent along its path is maximized.

During training, the new *Q* value, $Q^{\textrm{new}}$, must be calculated for each pair of state and action variables, which is done via the Bellmann equation [41]:

6 $$\begin{aligned}&Q^\textrm{new}(s_{t},a_{t}) \leftarrow Q(s_{t},a_{t}) \\&\qquad +\alpha \cdot \big [(r_{t} + \gamma \, \max _{a}Q(s_{t+1}, a)- Q(s_{t},a_{t}) \big ] \, .\nonumber \end{aligned}$$

Here, $s_t$ is the current state and $a_t$ the current action variable. The immediate reward $r_t$ belongs to taking action $a_t$ in state $s_t$, and $s_{t+1}$ is the new state reached. The term $\textrm{max}[Q(s_{t+1},a_t)]$ represents the maximum expected future reward for the new state variable $s_{t+1}$. The discount factor $\gamma$ determines how much the future reward is taken into account, and $\alpha$ quantifies the learning speed. The optimal values of both factors depend on the specific problem. In Appendix A.2, we shortly address this for our optimization tasks. One can show that defining an immediate reward suitable to the optimization task, the *Q* matrix will converge toward its optimal value by applying the recursion formula (6) [56].

We combine the deterministic choice of the action with the $\epsilon$-greedy method. The most rewarding action is only taken with probability $1-\epsilon$ and otherwise the action is chosen randomly. This prevents that the optimal path becomes stuck in a local minimum. We decrease $\epsilon$ with each episode according to $\epsilon = 0.5 (1 - i/i_\text {max})$, where $i_\text {max}$ is sufficiently large to guarantee that the *Q* matrix has converged and the phenomenological factor 0.5 guarantees the fastest convergence. Despite using the $\epsilon$-greedy method, in the following we will call this version also deterministic *Q* learning to distinguish it from *Q* learning under the presence of orientational thermal noise.

### Choice of state and action variables

To find the optimal navigation in a complex environment using tabular *Q* learning, information about the position of the particle is required. The *x*, *y* coordinates are often used to define the state of the particle [14, 33]. However, having information about the position is certainly difficult for a microswimmer to realize, when video microscopy and some external information processing are not available [14]. It seems more realistic and easier for microswimmers to sense the direction and distance to a target using, e.g., a magnetic field, in which magnetotactic bacteria experience a torque, a light field in combination with phototaxis, or olfactory sensing [57–59]. Also sensing a chemical field is an option; however, the concentration field can be distorted by the surroundings. Thus, compared to our previous work [33], to describe the state of the active particle, we perform a coordinate transformation $(x,y) \rightarrow (\rho ,\varphi )$, where $\rho$ is the distance and $\varphi$ the direction angle to the target (see Fig. 1). Reducing the dimension of the state space by omitting $\rho$ or $\phi$ is not possible within the *Q*-learning formalism since then the required action, which depends on the position of the active particle, cannot unambigously predicted.

In the following, we will use these alternative state variables and *Q* learning to solve the navigation problem. As we mentioned previously, the tabular *Q*-learning method creates a matrix with finite number of elements. Thus, the state space consists of a set of 2*N* discrete state variables, for which we choose:

7 $$\begin{aligned}&\text {distance:}\qquad \rho _i \in [\rho _1 = 0,\rho _2,...,\rho _N] \end{aligned}$$

8 $$\begin{aligned}&\text {direction:}\qquad \varphi _i \in [ \varphi _1 = 0,\varphi _2,...,\varphi _N] \, . \end{aligned}$$

The active particle moves continuously in space. We assign its distance $\rho$ and direction angle $\varphi$ to the discrete values $\rho _i$ and $\varphi _i$, when $\rho$, $\varphi$ fall within the intervals $[\rho _i -\Delta \rho /2, \rho _i + \Delta \rho /2]$ and $[\varphi _i -\Delta \varphi / 2, \varphi _i + \Delta \varphi /2]$, respectively. Here, $\Delta \rho = (\rho _N - \rho _1) / (N-1)$ and $\Delta \varphi = 2\pi /N$, and for the end points $i=1,N$ only half of the intervalls are taken. Equally, the action variable is the discretized orientation angle of the active particle, for which we always take eight values:

9 $$\begin{aligned} \text {orientation:}\qquad \theta _i = (i-1)\frac{\pi }{4}\, , \quad i=1,...,8 \, . \end{aligned}$$

Now, for the discrete state variables an action variable is selected according to the *Q* matrix, so the active particle changes its orientation from $\theta _t$ to $\theta _{t+1}$. Then, the active particle moves in continuous space. Using Eq. (1), it reaches the position

10 $$\begin{aligned} {\textbf {r}}^{\textrm{new}} ={\textbf {r}} + {\textbf {v}}\ \Delta t \, , \end{aligned}$$

for which we calculate the new distance $\rho$ and direction angle $\varphi$. The procedure is repeated until the particle arrives at the target, meaning $\rho < \Delta \rho / 2$, and one training episode is completed. If the target is not reached after 500 time steps, the episode is stopped and a negative reward of $R=-10$ is generated. The episodes are repeated until the *Q* matrix converges and the optimal solution is found. In practice, we choose $i_{\textrm{max}}$ from the $\epsilon$-greedy method sufficiently high to achieve convergence.

In case that we include thermal noise in the orientation vector $\textbf{e}$ or a flow field with nonzero vorticity $\omega _D$, we first let the orientation angle evolve according to Eq. (5) and then the translational step according to Eq. (10) is performed. Note also that we do not use here the $\epsilon$-greedy method, since thermal noise naturally brings in some randomness.

### System parameters

In units of the distance *L* between start and target, the quadratic system extends in *x* and *y* direction from $- 0.75$ to 0.75. Start and target are placed on the *x* axis at $-0.5$ and 0.5, respectively. We use $N=43$ discrete values for $\rho$ and $\varphi$ with the maximum distance $\rho _N = 1.4577$ and the resolution $\Delta \rho = 0.0343$. This guarantees that all positions from the target are reachable. Typically, the time step is $\Delta t = 0.0375$. However, for the case of crossing a uniform flow (Sect. 3.3) and the Poiseuille flow (Sect. 3.4), we need to increase it with the strength of the flow, and for the case of a swirling flow (Sect. 3.5), $\Delta t$ needs to decrease for stronger swirls.

The time step should roughly be adjusted such that during one time step the particle can move from one grid element to a neighboring element. Choosing it smaller does not bring any improvement. On the contrary, the *Q*-learning algorithm has to perform numerous steps to move the particle forward, without reaching other state variables, which can considerably slow down the whole learning process. For the two cases of the uniform and Poiseuille flow, the time step needs to be increased to have a noticeable motion across the flow, while for the swirling flow $\Delta t$ needs to decrease in order to properly approximate the trajectory.

To start the training, the *Q* matrix must be initialized. Here, we set all entries uniformly to $Q(s, a) = 100$ [41]. An important point is how the rewards $r_t$ for the different actions within *Q* learning are chosen. Since we want to minimize travel time *T*, each step of duration $\Delta t$ receives a reward $r_t = - \Delta t$. So the negative reward is smallest if the number of steps is minimized. Reaching the target gives a reward of 100, and if the particle crosses the border of the system, a large negative reward of $-10$ is used to strongly penalize this action. Furthermore, when performing such a move, the particle is placed back to the location, where the border was crossed. This procedure corresponds to implementing a hard-core repulsion from the border, while the negative reward signals the particle within the learning phase to avoid such steps. Without such a negative reward the learning takes longer. The learning speed and discount factor are chosen as $\alpha = 0.9$ and $\gamma = 0.7$, respectively. In Appendix A.2 we present a parameter study to determine the optimal values for $\alpha$ and $\gamma$. Finally, for implementing the $\epsilon$-greedy method, $i_{\textrm{max}} = 5000$ is often sufficient for the travel time to converge, and we never need to go beyond $i_\textrm{max} = 20000$.

**Fig. 2:**
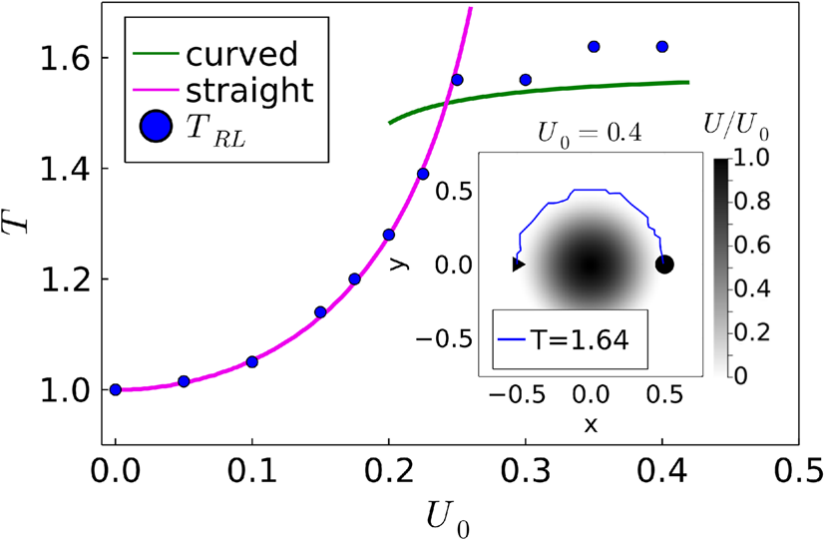
Shortest travel time *T* versus barrier height $U_0$ either for crossing the barrier of a Mexican hat potential on a straight path (magenta) or moving around it (green). The blue dots indicate the results of *Q* learning. The inset shows a grayscale representation of the potential and the optimal path determined with *Q* learning for $U_0 = 0.4$

## Results

We now present results for a few types of drift-velocity fields, which either derive from a potential or are due to an imposed flow field.

### Potential barrier and well

In our earlier work [33], we determined the fastest path, when there is a potential barrier between the start and target. We modeled the barrier using the Mexican hat potential without brim,

11 $$\begin{aligned} U = \left\{ \begin{array}{c l} 16 U_0 (r^2 - 1/4)^2 , &{} r \le 1/2 \\ 0 , &{} \text {otherwise} \end{array} \right. \, , \end{aligned}$$

where *r* is the radial distance to the center. At $r=0$, the potential has a maximum with height $U_0$, and on the ring $r=1/2$ it is zero with horizontal tangent. The maximum potential force $-\nabla U$ is at $r = 1 / 2\sqrt{3}$ with $\vert \nabla U \vert = 16U_0 / 3\sqrt{3}$. The inset of Fig. 2 shows a grayscale representation of the potential.

The potential force can be incorporated in Eq. (1) by choosing $\textbf{v}_D(\textbf{r}) = -\nabla U$. As we already discussed in detail in Ref. [33], the variation $\delta T = 0$ identifies the straight path over the potential barrier as optimal until $U_0 =0.24$, where the curved path around the barrier becomes faster (see main plot of Fig. 2). There is also a regime, where both paths are locally stable. *Q* learning is able to identify the optimal paths, as the blue dots in Fig. 2 show. For the curved path (an example is shown in the inset), the minimum travel time is better approximated than compared to Ref. [33] since we allow the active particle to move freely in space and not just on a grid.

**Fig. 3:**
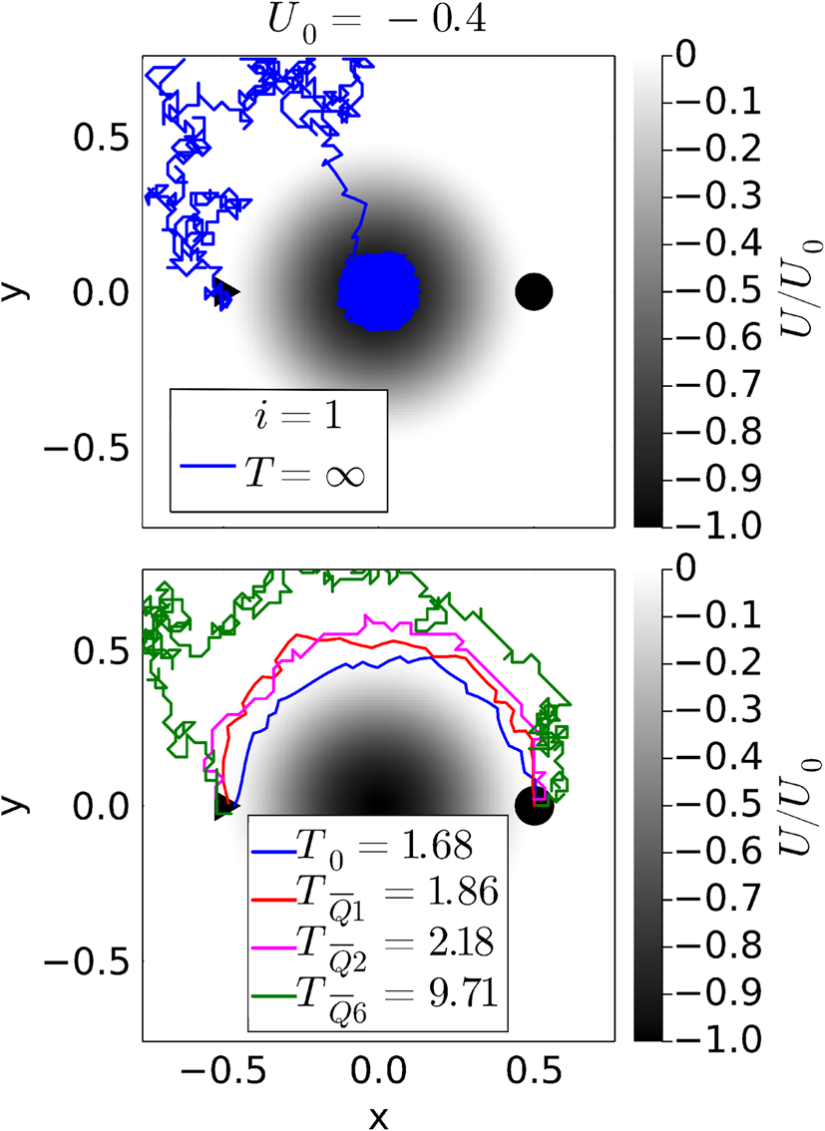
Potential well with $U_0 = -0.4$. Top: In the first episode ($i=1$), the active particle becomes trapped in the well and the learning ended after $T=500$. The current action receives a negative reward of $-10$. Bottom, blue trajectory: After $i_\textrm{max}$ = 50,000 the active particle has learned to avoid the well and move around it. Other trajectories: Example trajectories for applying an averaged *Q* matrix under noise. The index *n* in the corresponding travel time $T_{\overline{Q}n}$ refers to the strength of the orientational thermal noise, $n=\sqrt{D_R L/v_0}$

By choosing a negative $U_0$, the potential barrier becomes a well. The phenomenology of the paths is the same as for the barrier. However, there is one difference. If the depth of the well is below $U_0 =-3\sqrt{3}/16 = -0.325$, the active particle cannot escape the well. This is illustrated for $U_0 = -0.4$ in Fig. 3, top, where we show the trajectory for the first episode. It is stopped after a travel time of $T=500$ and the current action receives a large negative reward of $-10$. The episodes are repeated, and obviously the active particle learns to avoid the well since it ultimately finds the optimal path around the potential well (Fig. 3, bottom). The blue curve with travel time $T_0$ refers to zero noise. However, since the active particle needs to explore and learn to avoid the “forbidden” region of the well, a larger $i_\textrm{max} = 50,000$ is necessary. This example shows very clearly that negative results of not reaching the target also contribute to the learning process of the active particle.

We add a final note. The policy for the optimal path is encoded in the *Q* matrix. However, this matrix contains more information. One can place the active particle at any location within the system, provided this location has been visited before in the learning phase, and the *Q* matrix will guide it to the target. However, in general, the path will not be the fastest. We have checked this for the potential barrier.

### Learning with noise

Orientational noise can be included during different stages of *Q* learning, which we explore here for the potential barrier and well. One can include noise while learning the optimal paths and when applying the optimal policy. In addition, one can vary the noise strength.

#### Potential barrier

**Fig. 4:**
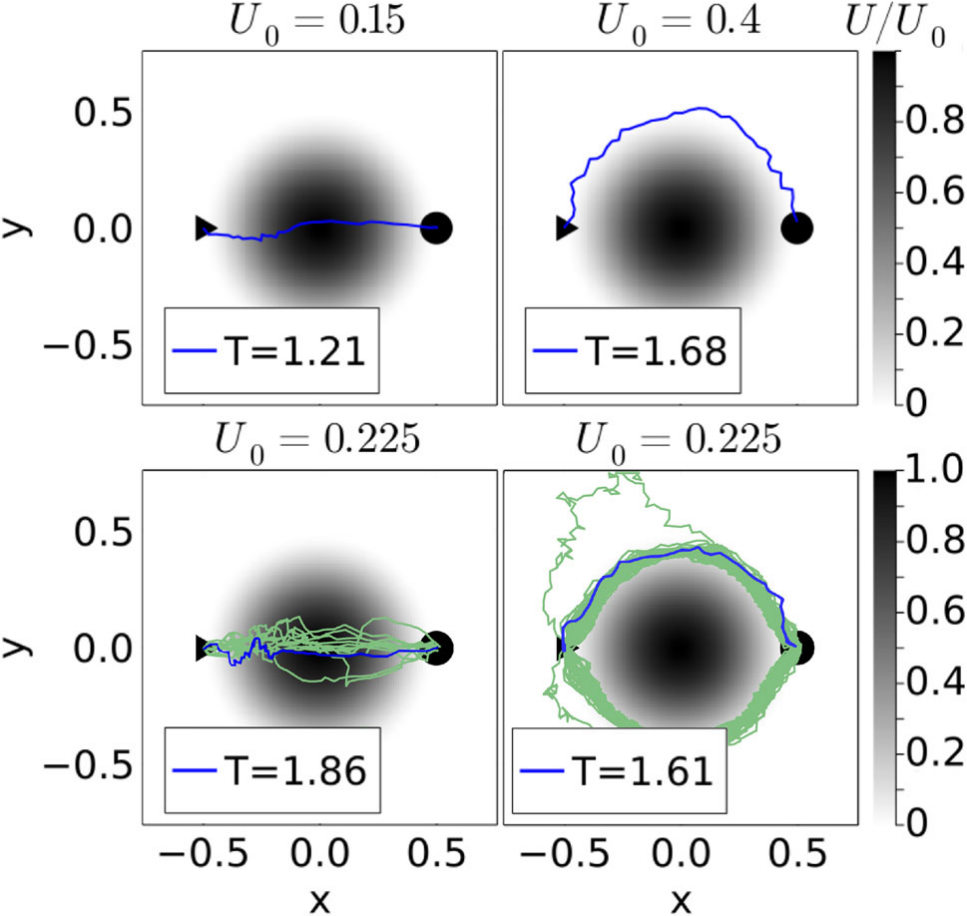
Orientational noise does not prevent learning the optimal paths. Top: Straight and curved paths for $U_0=0.15$ and $U_0=0.4$, respectively. Bottom: At $U_0 = 0.225$ both paths are realized. In light green, 100 paths are shown. Each of them results from learning the optimal path under noise

We start with exploring the influence of noise during learning. The prefactor $\sqrt{D_R L/v_0}$ in Eq. (5) with $\omega _D =0$ was set to one to have a noticeable change of particle orientation $\textbf{e}$ during the time step $\Delta t$. For example, for a random number of $W_\theta = 1$ the change in orientation angle, $\theta ^\text {new} - \theta$, is $11^\circ$. Nevertheless, the active particle learns to move accross the barrier and around it as Fig. 4, top illustrates for low and high $U_0$. However, the learned trajectories are noisy, which also increases the travel time compared to the deterministic case. Interestingly, at $U_0 = 0.225$ close to the point ($U_0 = 0.24$) where the absolute stability switches from the straight to the curved path, we observe both types of paths as Fig. 4, bottom shows. Thus, noise causes the optimization process to converge into different local minima. Interestingly, the travel time of the straight path is more strongly affected by orientational noise compared to the curved path and, therefore, its value is more strongly enhanced. This makes sense since the acting potential forces drive the particle away from the optimal path. In light green, we plot 100 trajectories each determined from a separate learning run. They show that learning under noise can reproduce the two types of trajectories; only a few of them deviate more strongly. Alternatively, one can also take a specific learned *Q* matrix and apply it several times under noise (not shown in Fig. 4). While the curved paths are reproduced, now the “straight paths” are strongly distorted and nearly cover the whole region of the potential barrier; again because noise affects them more strongly.

**Fig. 5:**
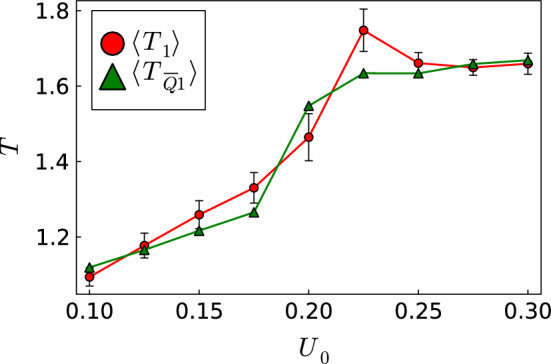
Mean travel time $\langle T_1 \rangle$ (red circles) and standard deviation $\Delta T_1$ (error bars) plotted versus $U_0$ from *Q* learning in the presence of orientational thermal noise during the learning phase. Mean travel time $\langle T_{{\overline{Q}}1} \rangle$ (green triangles) for applying an averaged *Q* matrix under noise. The lines are a guide to the eye

To quantify our observations further, we then took the 100 *Q*-learning runs for the same $U_0$ and calculated the mean of the travel time, $\langle T_1 \rangle$, and its standard deviation $\Delta T_1$, where the index 1 refers to the noise strength $\sqrt{D_R L/v_0} = 1$. Both quantities are plotted versus $U_0$ in Fig. 5. At small and high $U_0$, the mean travel time behaves as the deterministic value in Fig. 2. It increases with $U_0$ for the straight path and levels off at high $U_0$ for the curved path. Similarly, the standard deviation increases for small $U_0$ and it is small for large $U_0$. Hence, noise does not cause large variations. Interestingly and different compared to the deterministic value, the mean travel time becomes maximal at $U_0 = 0.225$ where both path types are observed, especially the noisy straigth path with longer travel time. Accordingly, close to this value the standard deviation has a maximum.

As an alternative approach dealing with noise, we performed 10 *Q*-learning runs and used the averaged optimal *Q* matrix to run 100 trajectories in the presence of orientational noise. Note in the following the averaged *Q* matrix is abbreviated as $\langle Q \rangle$ and when hinting to it in an index as $\overline{Q}$. The mean travel time $\langle T_{{\overline{Q}}1} \rangle$ (green triangles in Fig. 5) does not deviate strongly at small and large $U_0$ from $\langle T_1 \rangle$, where $T_1$ was calculated for each newly learned trajectory. Only around $U_0 = 0.225$ the deviation is stronger and the green curve misses the peak. The reason is that already at $U_0 = 0.225$ the occurence of straight paths under orientational noise is rare and the 10 learned *Q* matrices, from which we determine the mean $\langle Q \rangle$, belong to the curved path.

Finally, we add that to achieve these results, we did not use the $\epsilon$-greedy method. Thus, also thermal noise helps in finding optimal trajectories. We also checked that including the $\epsilon$-greedy method did not change our results significantly.

#### Potential well

Of course, increasing the strength of the orientational thermal noise, $\sqrt{D_R L/v_0}$, the “optimal paths” and travel times deviate more and more from their deterministic values. To illustrate this, we consider the potential well with $U_0=-0.4$ (Fig. 3, bottom), where the active particle cannot leave once it moves too close to the center. With increasing noise, the learned *Q* matrices differ strongly from each other. So we decided to use a mean optimal *Q* matrix averaged over 10 learning runs under the same noise strength. Figure 3, bottom shows example trajectories when applying $\langle Q \rangle$ for different noise strengths, where the index *n* in $T_{\overline{Q}n}$ refers to $n=\sqrt{D_R L/v_0}$. One already recognizes, as a strategy for not becoming trapped in the well, the particle keeps a larger distance to the well with increasing noise.

**Fig. 6:**
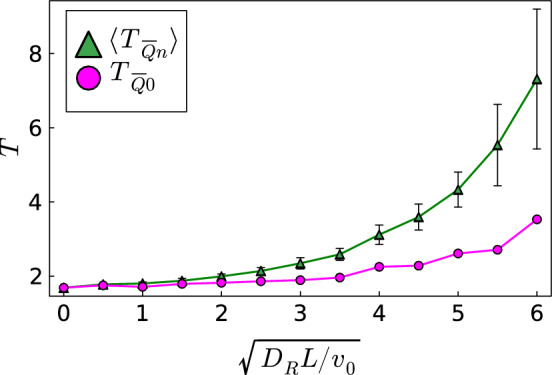
Mean travel time $\langle T_{\overline{Q}n} \rangle$ (green triangles) and standard deviation (error bars) plotted versus strength of the orientational thermal noise, $\sqrt{D_R L/v_0}$, for the potential well with $U_0 = -0.4$. Under noise, 10 optimal *Q* matrices are determined and the average $\langle Q \rangle$ is applied 100 times to determine $\langle T_{\overline{Q}n} \rangle$. The time $T_{\overline{Q}0}$ (magenta circles) refers to $\langle Q \rangle$ applied without noise. The lines are a guide to the eye

This is illustrated quantitatively in Fig. 6. We applied $\langle Q \rangle$ 100 times and then plot $\langle T_{\overline{Q}n} \rangle$ and its standard deviation, represented as error bars, versus the strength of the orientational noise. One realizes noise does not only have the effect of moving the trajectory further away from the center and thereby increasing the travel time. In addition, noise lets the trajectory become more irregular (see Fig. 3, bottom). How much this contributes to the travel time is clarified by the red circles in Fig. 6. Here, we apply $\langle Q \rangle$ without noise. So $T_{\overline{Q}0}$ shows the pure effect of the particle, which needs to avoid the potential well when learning under noise to reach the target.

### Crossing uniform flow

So far, we used a potential force acting on the active particle. Now, we put the active particle in a uniform flow field along the *y* axis with strength *k*, $\textbf{v}_D = k\,\textbf{e}_y$, so that the total velocity of the active particle becomes

12 $$\begin{aligned} \textbf{v}=\textbf{e} + k\,\textbf{e}_y \, . \end{aligned}$$

**Fig. 7:**
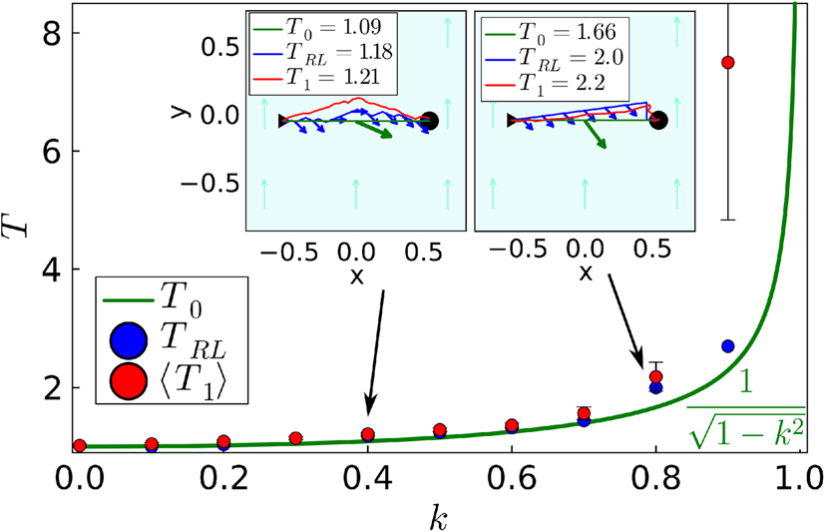
Optimized travel time *T* versus strength *k* in a uniform flow field. Green line: from analytic optimization. Blue dots: from deterministic *Q* learning. Red dots: mean of 100 *Q*-learning runs under noise, and error bars indicate the standard deviation. Insets: Examples of learned trajectories for $k=0.4$ and 0.8, respectively. Green: analytic minimum, blue: deterministic *Q* learning, red: *Q* learning under noise. The green arrow and the blue arrows indicate the respective particle orientations for the first and second case

The Euler-Lagrange equation corresponding to the variation of the travel time, $\delta T = 0$, can be formulated and solved analytically. The calculation is a bit lengthy but straightforward. It always gives the straight path between start and target as optimal as shown for two examples in the insets of Fig. 7 (green lines). For increasing flow strength *k*, the orientation vector $\textbf{e}$ has to tilt more and more against the imposed flow to avoid that the active particle drifts downstream. Indeed, one finds for the optimal travel time

13 $$\begin{aligned} T = \frac{1}{\sqrt{1-k^2}} \, , \end{aligned}$$

which diverges for $k \rightarrow 1$. Here, the active particle points fully against the flow and there is no component of the swimming velocity left to cross the flow field along the *x* direction. The blue dots in Fig. 7 show the results of the optimization from deterministic *Q* learning and for two cases, we show the paths (blue lines) and the particle orientations (blue arrows) in the insets. The straight path along the *x* direction is not realized since the particle orientation $\textbf{e}$ can only assume eight discrete orientations. Note, to perform *Q* learning, we needed to increase the time step $\Delta t$ from 0.0375 to 0.1 with increasing *k*.

In a last step, we include orientational thermal noise when performing 100 *Q*-learning runs. Including the time step, the noise in Eq. (5) is determined by $\sqrt{(D_R L/v_0) \Delta t}$. To apply the same noise during a time step, we always choose $(D_R L/v_0) \Delta t = 0.0375$. Thus, when increasing $\Delta t$, we reduce $D_R L/v_0$ accordingly. The red dots in Fig. 7 show the mean travel time $\langle T_1 \rangle$ from the 100 *Q*-learning runs, and the error bars indicate the standard deviation. Until $k=0.8$, no strong deviation from deterministic *Q* learning is observed and the red trajectories in the insets show typical examples. In contrast, the trajectories for $k=0.9$ strongly deviate from each other, which results in a strongly increased $\langle T_1 \rangle$ with a large standard deviation. The reason is that the particle orientation is nearly antiparallel to the flow field, so orientational fluctuations can cause the particle to move away from the target instead of toward it. These excursions result in a strongly increased $\langle T_1 \rangle$.

### Crossing Poiseuille flow

**Fig. 8:**
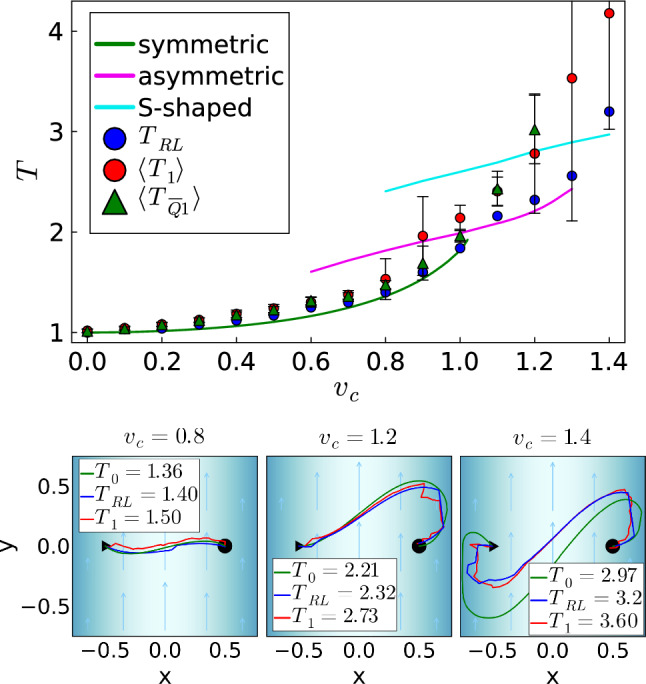
Top: Optimized travel time versus strength $v_c$ of the Poiseuille flow. Solid lines: numerical minimization gives three trajectory types. Blue dots: from deterministic *Q* learning. Red dots: mean of 100 *Q* learning runs under noise, and error bars indicate the standard deviation. Green triangles: $\langle T_{\overline{Q}1} \rangle$ from applying a mean *Q* matrix under noise. Bottom: From left to right, examples of the three trajectory types are indicated: symmetric, asymmetric, and S-shaped. Green: numerical minimization, blue: deterministic *Q* learning, red: *Q* learning under noise

In our next example, we implement a Poiseuille flow field along the *y* axis with zero velocity at the boundaries of our typical system geometry, $x=\pm 3/4$. So the *y* component of the velocity field $\textbf{v}_D$ becomes:

14 $$\begin{aligned} v_y=v_c \Big [1- \Big (\frac{4}{3}x\Big )^2 \Big ] \, , \text { where } \omega _D= -\frac{16}{9}v_c x \end{aligned}$$

describes the rotational velocity experienced by the active particle due to the nonzero flow vorticity. To keep the problem simple, we do not implement any hydrodynamic interactions with the bounding walls. Again, we look for the fastest trajectory between starting and target positions located at $-0.5$ and 0.5 on the *x* axis, respectively.

We first explore the numerical minimization of the travel time $T_0$, which gives three types of trajectories that correspond to the three branches (solid lines) in Fig. 8, top. As for the potential barrier, these branches overlap when tuning the flow strength $v_c$ indicating up to three local minima. For $v_c \lessapprox 1$ the optimal trajectory is curved symmetrically about the *x* axis (green trajectory in Fig. 8, bottom left). Thus, close to the starting and target positions, where the flow velocity field is smaller, the active particle can swim upstream, while it drifts downstream in the center. Then, for $v_c > rapprox 1$ the shape becomes asymmetric (green trajectory in Fig. 8, bottom middle) since the active particle needs to explore the slow flow close to the wall for being able to reach the target. Similarly, also the trajectory mirrored at the center exists. Ultimately, at $v_c \approx 1.3$ an S-shaped trajectory is the optimum in travel time (green trajectory in Fig. 8, bottom right). The slow flow at both walls is used to swim upstream in order to compensate for the downstream drift in the center.

Now, reinforcement learning without orientational noise (blue dots in Fig. 8) nicely reproduces the optimal travel times, although at $v_c = 1.4$ the trajectories differ (compare green and blue trajectories in Fig. 8, bottom right). To perform *Q* learning, we needed to increase the time step $\Delta t$ from 0.0375 to 0.08 with increasing $v_c$.

**Fig. 9:**
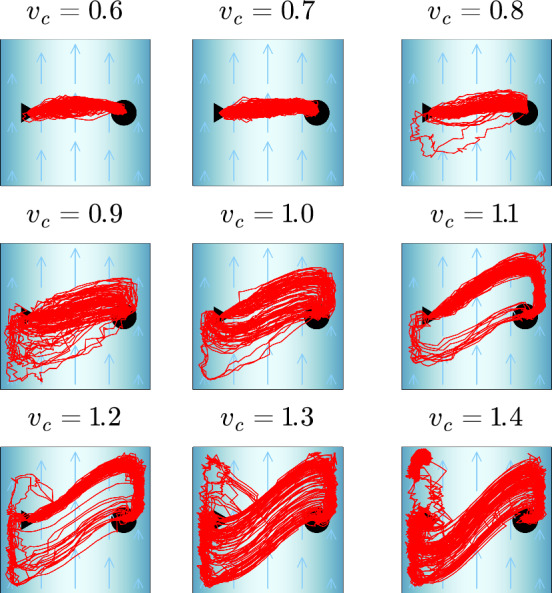
For several $v_c$, all the 100 learned trajectories resulting from *Q* learning under noise are shown

In a next step, we again include orientational noise and perform an average over 100 *Q*-learning runs to determine a mean travel time. As for the uniform flow, we keep the noise per time step constant by choosing $(D_R L/v_0) \Delta t = 0.0375$ in Eq. (5). The red dots in Fig. 8, top show the mean travel time $\langle T_1 \rangle$ and the error bars indicate the standard deviation. Up to $v_c = 0.8$, $\langle T_1 \rangle$ agrees well with the numerical minimization ($T_0$) and deterministic *Q* learning ($T_{RL}$). Also the different trajectories vary around the symmetric path (top row in Fig. 9). At $v_c = 0.9$, a mixture of symmetric and asymmetric trajectories (Fig. 9, middle row) results in a stronger deviation from $T_0$ and $T_{RL}$ and a larger standard deviation. Then, at $v_c=1.1$ only the asymmetric trajectory type is realized, which at $v_c=1.3$ starts to also develop the S-shaped type. However, since the trajectories become more and more irregular, $\langle T_1 \rangle$ deviates more strongly from $T_0$ and $T_{RL}$ with large standard deviations.

**Fig. 10:**
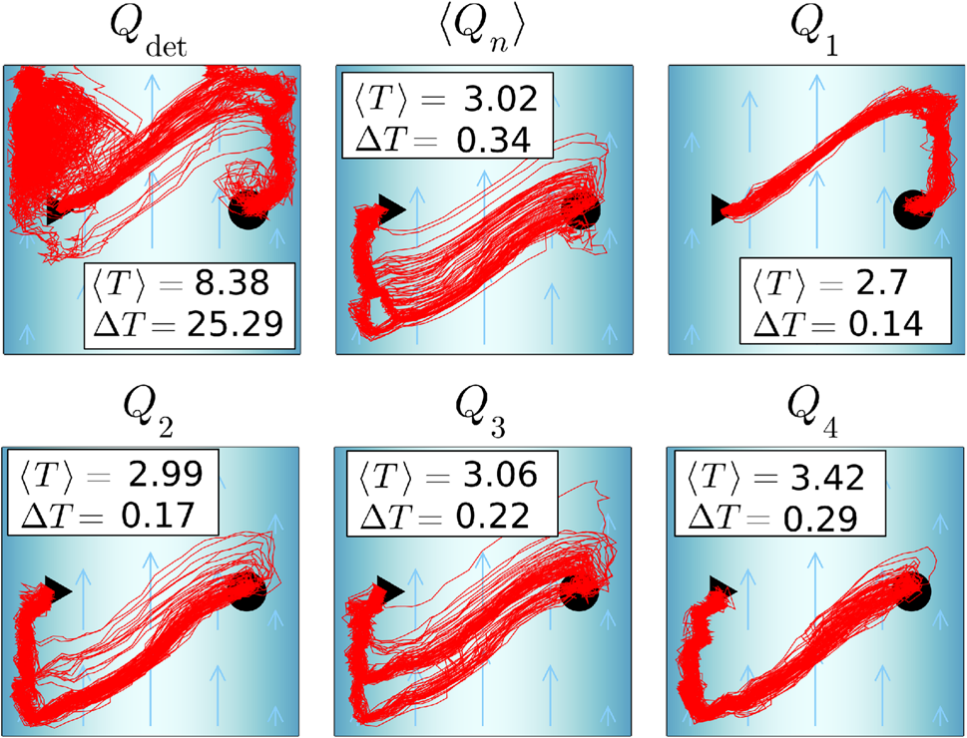
Each plot shows 100 trajectories that result from applying different types of *Q* matrices under noise at $v_c = 1.2$. $\langle Q_n \rangle$: mean *Q* matrix from 10 *Q* learning runs under noise. $Q_1$–$Q_4$: examples of *Q* matrices learnt under noise. $Q_\text {det}$: deterministic *Q* matrix

**Fig. 11:**
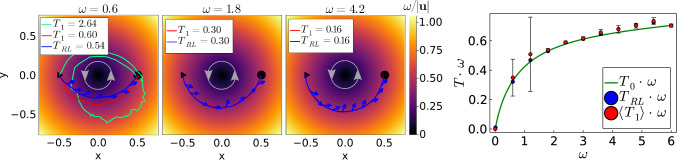
Swirling flow field. Left: Three examples of optimal paths for increasing swirling strength $\omega$. Blue line: from deterministic *Q* learning and blue arrows indicate the particle orientation. Red line: trajectory learned in the presence of noise. Green: The particle misses the target and has to circle once around the center. The gray circles indicate the direction of the flow. Right: Travel time *T* times $\omega$ plotted versus $\omega$. Green line: from numerical minimization. Blue dots: from deterministic *Q*-learning. Red dots: mean of 100 *Q*-learning runs under noise, and error bars indicate the standard deviation

In the end, we address the question how optimal policies encoded in learned *Q* matrices reproduce the learned trajectories when applied under noise. In Fig. 10, we compare the outcome for different types of learned *Q* matrices for flow strength $v_c = 1.2$, which we apply 100 times. At $v_c=1.2$, the optimized travel time is $T=2.21$. Interestingly, applying $Q_\text {det}$ (the optimal deterministic *Q* matrix) produces trajectories that make long detours under noise. Thus, the mean travel time $\langle T \rangle$ deviates strongly from the ideal value and the standard deviation is large. Taking a mean of 10 *Q* matrices learned under noise, $\langle Q_n \rangle$, gives trajectories without large detours, and the mean travel time is well below the one for $Q_\text {det}$. Even the single $Q_n$ matrices, when applied under noise, are more successful than $Q_\text {det}$ as the examples for four *Q* matrices in Fig. 10 show. These results suggest that *Q* matrices learnt under noise or their average are less volatile to noise than the deterministic *Q* matrix, when using them. In Fig. 8, the green triangles indicate the mean travel time for $\langle Q_n \rangle$. Up to $v_c = 0.7$, there is not much difference between the differently determined travel times. Interestingly, between $v_c = 0.8$ and 1.0, the mean *Q* matrix provides a better travel time than the average over 100 *Q*-learning runs. However, for $v_c=1.2$ this is no longer true and for $v_c = 1.3$ and 1.4 some of the trajectories did not reach the target, so we do not provide a mean travel time here.

### Crossing swirling flow

As a last example, we investigate the case, where the active particle needs to cross a swirling flow on its way to the target. We consider

15 $$\begin{aligned} \textbf{v}_D=\dfrac{\omega }{r} \textbf{e}_{\varphi } \, , \end{aligned}$$

which has zero vorticity ($\textrm{curl} \textbf{v}_D = 0$) so that the particle orientation $\textbf{e}$ is not rotated by the flow. Figure 11, left shows three optimal paths determined from deterministic *Q* learning for increasing flow strength $\omega$. One observes that for smaller $\omega$ the active particle crosses closer to the center, because here flow is larger, which helps to minimize travel time. The self-propulsion is needed to cross the circular streamlines in order to reach the target. For increasing $\omega$ and thus increased drifting, the active particle has to stay closer to the streamline of the target for being able to reach it. For large $\omega$, self-propulsion can more and more be neglected against drifting and the active particle moves on the half circle connecting start and target. This is approximately the case for $\omega = 4.2$.

The green graph in Fig. 11, right, a numerical minimization of the travel time confirms this view. For small $\omega$ the ideal path is nearly straight and $T \approx 1$ is constant or $T \omega$ is linear in $\omega$. For large $\omega$, $T \omega$ should tend to $\pi /4 = 0.786$, but the convergence is rather slow. The results from reinforcement learning without orientational noise (blue dots) agree rather well with the green curve. When just plotting *T* versus $\omega$, they nicely fall on top of each other. Small deviations become enlarged when plotting $T \omega$. To arrive at the results for $\omega \ge 1.5$, we increased the number of action orientations from 8 to 16 and successively decreased the time step $\Delta t$ to 0.001. The reason is that at larger flow strengths one has to fine-tune the position and orientation of the active particle to hit the target; otherwise, the particle needs to circle around the center to make another attempt.

Again we introduce orientational noise and perform 100 *Q* learning runs, while keeping the factor governing noise at $(D_R L/v_0) \Delta t = 0.0375$. At $\omega = 1.8$ and beyond, the mean travel times $\langle T_1 \rangle$ (red dots in Fig. 11, right) nicely agree with deterministic *Q* learning, the standard deviations are small, and the trajectories fall on top of each other (Fig. 11, left). However, at $\omega =0.6$ and 1.2 large standard deviations occur. They are due to rare events, where the particle does not hit the target and therefore needs to circle around once to reach it. An example (green trajectory) is given for $\omega =0.6$ in Fig. 11, left.

## Conclusion

In this article, we considered a smart active particle that can sense the distance and direction to a target. We used *Q* learning to demonstrate how the particle learns by itself to navigate on the fastest path in different potential landscapes and flow fields. In parallel, we also solved the optimization problem using variational calculus to show how well *Q* learning works. Our idea is that sensing distance and direction as state variables is easier to realize with a smart active particle than sensing the position.

First, we considered a potential barrier as in our previous work [33], but now the learned paths are closer to the optimal path since the active particle moves continuously in space instead of only occupying grid points of a square lattice. Furthermore, as action variables we employ eight orientations instead of only four. We also considered a potential well, which was deep enough so that the active particle could become trapped in it once the trapping force exceeds a critical value. However, the particle indeed learns to avoid the trap and moves around it. This is an important feature when studying the optimal path in an arbitrary landscape.

Second, we demonstrated how the active particle crosses a uniform flow. The learned travel times agree well with the analytic result of the optimization. Third, crossing a Poiseuille flow is more challenging to evaluate. By numerical minimization of the travel time, we identify three types of trajectories: symmetric, asymmetric, and S-shaped. The second and third types occur at higher flow strengths and use the small flow velocity at the channel walls to move upstream in order to being able to cross the flow. Fourth, we also looked at a swirling flow. For small flow strengths, the active particle uses the larger flow velocities close to the center to arrive fastest at the target, while for larger flow strengths it has to stay on the circular flow line so that it does not miss the target. If this happens, the particle needs to circle around the center which increases the travel time.

Finally, for all the reported drift-velocity fields we evaluated the effect of orientational thermal noise during *Q* learning and when applying the optimal policy. Generally, noise does not prevent the active particle from learning optimal travel paths and to navigate to the target. Now the optimal path is noisy, which increases the travel time, and, of course, finding optimal paths depends on the strength of noise. Further general statements on the impact of noise are difficult. It rather depends on the specific problem and the trajectory to be learned. For example, for the potential well the learned trajectories run further away from the center with increasing noise to avoid that particles get trapped in the well. We add two further findings. First, when performing *Q* learning under noise for all drift-velocity fields, we identified the strategy to average over several *Q* learning runs. This works well if the optimized trajectories are well accessible by numerical minimization of the travel time or by deterministic *Q* learning. Second, when applying the learned optimal policy under noise, we made the interesting observation that a mean *Q* matrix works better than the deterministic *Q* matrix. The reason might be that the *Q* matrix averaged over several noisy learning runs has developed a better strategy to respond to random changes in the orientation compared to the deterministic *Q* matrix. Thus, our study also adds to the recent efforts to explore the stability of learned optimal strategies/policies [35, 51].

As a next step, we plan to study optimal navigation of active particles in more complex potential and flow landscapes using deep *Q* learning, which employs neural networks. This will also enable us to train the active particle to move optimally in a set of complex landscapes and then let it move in an unknown landscape.

## Figures and Tables

**Fig. 12:**
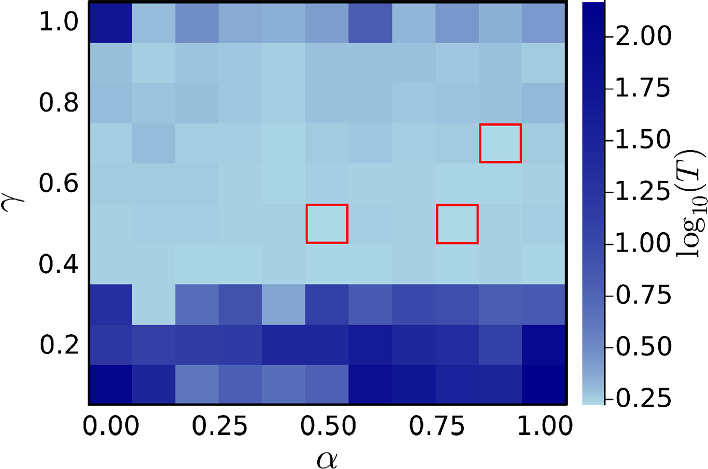
Heatmap for travel time *T* as a function of learning rate $\alpha$ and discount factor $\gamma$ for the active particle moving around the potential barrier with $U0=0.4$. $\epsilon =0.4$ is kept constant. The red boxes mark three minima for the travel time *T*

**Fig. 13:**
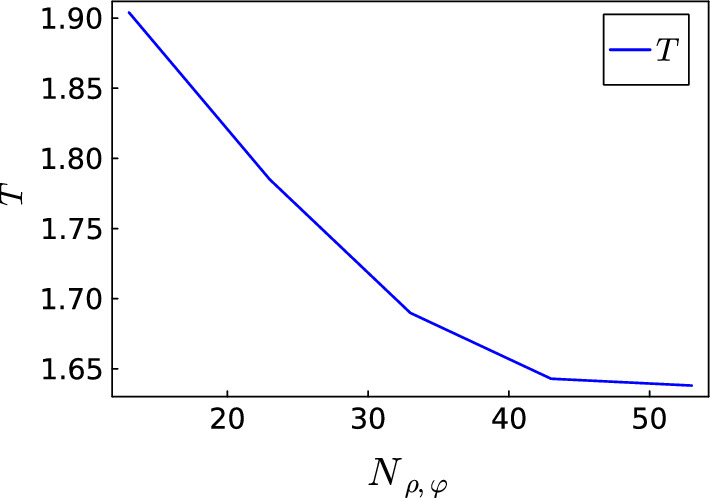
Travel time *T* plotted against the number of states $N_{\rho } = N_{\varphi }$ for the curved path around the potential barrier with $U_0 = 0.4$

## Data Availability

The datasets generated during and/or analyzed during the current study are available from the corresponding author on reasonable request.

## A Appendix

### A.1 Numerical minimization of the travel time.

The analytical solution of the Euler-Lagrange equations belonging to the variation of Eq. (2) can only be found for the uniform flow field used in Sect. 3.3. It is presented in Eq. (13). For the remaining force and flow fields, minimizing the functional of Eq. (2) requires a numerical approach. This is accomplished by discretizing Eq. (2) and employing numerical methods to minimize it.

The discretization approximates the trajectory by $N=200$ points $\textbf{r}(s) \rightarrow \textbf{r}_i(s)$, where *i* is the index of the *i*-th point. Additionally, the line element $ds_i$ and the tangent vector $\textbf{t}_i$ are approximated by

16 $$\begin{aligned} \textrm{d}s_i = \sqrt{({\textbf {r}}_{i+1}-{\textbf {r}}_{i})^2} \text { and } {\textbf {t}}_i = \dfrac{{\textbf {r}}_{i+1}-{\textbf {r}}_{i}}{\textrm{d}s_i}. \end{aligned}$$

Furthermore, using the discretized drift-velocity field ${\textbf {v}}_{Di} ={\textbf {v}}_D({\textbf {r}}_i)$ and the magnitude of the total velocity $v_i=v({\textbf {r}}_{i+1},{\textbf {r}}_i)$ from Eq. (3), the functional for the travel time *T* is approximated as

$$\begin{aligned} \int _{\textbf{r}_i}^{\textbf{r}f} \textrm{d}t \longrightarrow \sum _{i=1}^{N-1} \frac{\textrm{d}s_i}{v_i} \, . \end{aligned}$$

This expression can be minimized by using the optimization package from Julia, Optim.jl, where different solver algorithms can be used. Due to difficulties in calculating the Hessian for the Poiseuille flow, we successfully employed a gradient-based algorithm such as Conjugate Gradient, where the parameters of the algorithm needed to be tuned. Nevertheless, to optimize the travel time for the Poiseuille flow, it was necessary to use the results from reinforcement learning as initial trajectories. This approach provides a better starting point for the optimization process and improves the convergence of the solver algorithm.

In general, the *x*-coordinate in the optimization problem is treated as a variable to be determined. However, for simpler cases like the Mexican hat potential, it is sufficient to choose equally spaced *x* coordinates and to keep them fixed during the minimization. This reduces the complexity of the optimization problem and makes the solver more efficient in finding the optimal solution.

### A.2 Learning rate and discount factor

We will shortly discuss the procedure for determining the optimal values for the learning rate $\alpha$ and the discount factor $\gamma$. In Fig. 12, we demonstrate how choosing the parameters influences the travel time *T*. We created a heatmap, where $\gamma$ versus $\alpha$ is plotted, while the variation in color represents the logarithmic values of the travel time *T*. The study was performed for the potential barrier with $U_0=0.4$. The inset in Fig. 2 shows the learned trajectory. We do not consider values for $\gamma < 0.1$, otherwise the travel time diverges toward infinity. We can clearly see that the interval $\gamma \in [0.4,0.9]$ is the most suitable. In this interval, we can identify several minima for the travel time marked as red boxes in Fig. 12. Since we deal with fully deterministic environments, a high learning rate can be chosen to achieve a fast convergence of the *Q* function [41]. Thus, we choose our parameters as $\gamma =0.7$ and $\alpha =0.9$.

### A.3 State space resolution

In our reinforcement learning study, the state space is defined by the angle $\varphi$ and distance $\rho$ to the target, with both variables discretized into 43 values each. Although the resolution of this approach might seem equivalent to a 43x43 quadratic grid, there are some factors that add complexity to the problem.

First, the linear resolution along the angular direction is not uniform across the state space, as it decreases for larger $\rho$ values. This leads to unequal tile sizes, with linear dimensions as large as 0.221 in the angular direction at the edge of the state space compared to 0.033 along the radial direction. As a result, the active particle needs to perform multiple actions, depending on the time step $\Delta t$, before transitioning to another state. Conversely, around the target, the angles are very small, causing a large number of states to be concentrated in that area. This non-uniform distribution of states poses challenges for the reinforcement learning process.

Second, due to the definition of the state space in relation to the quadratic shape of the system, ca. 50 % of the potential states are never visited by the active particle. Only 957 of the 1849 possible states are actually encountered during the learning process.

To investigate the impact of the state space resolution, we examined how the number of states $N_{\rho ,\varphi }$ along the $\rho$ and $\phi$ direction influences the travel time *T*. We choose $N_{\rho }=N_{\varphi }$ and plot in Fig. 13 travel time *T* versus $N_{\rho , \varphi }$ for the special case of the potential barrier at $U_0=0.4$, where the curved path is realized. We observe that the travel time decreases for increasing $N_{\rho ,\varphi }$. It is almost saturated at $N_{\rho ,\varphi }=43$, which we use in our investigation. Consequently, a further increase in $N_{\rho ,\varphi }$ is not necessary to guarantee the applicability of *Q* learning.
